# New insights into the wheat chromosome 4D structure and virtual gene order, revealed by survey pyrosequencing

**DOI:** 10.1016/j.plantsci.2014.12.004

**Published:** 2015-04

**Authors:** Marcelo Helguera, Máximo Rivarola, Bernardo Clavijo, Mihaela M. Martis, Leonardo S. Vanzetti, Sergio González, Ingrid Garbus, Phillippe Leroy, Hana Šimková, Miroslav Valárik, Mario Caccamo, Jaroslav Doležel, Klaus F.X. Mayer, Catherine Feuillet, Gabriela Tranquilli, Norma Paniego, Viviana Echenique

**Affiliations:** a Estación Experimental Agropecuaria Marcos Juárez, Instituto Nacional de Tecnología Agropecuaria (INTA), Marcos Juárez, Córdoba, Argentina; b Instituto de Biotecnología, Centro Investigación en Ciencias Veterinarias y Agronómicas (CICVyA) INTA, Hurlingham, Buenos Aires, Argentina; c Consejo Nacional de Investigaciones Científicas y Técnicas (CONICET), Argentina; d The Genome Analysis Centre (TGAC), Norwich Research Park, Norwich NR4 7UH, UK; e MIPS/IBIS, Helmholtz Zentrum München, 85764 Neuherberg, Germany; f CERZOS (Centro de Recursos Naturales Renovables de la Zona Semiárida), (CCT-CONICET-Bahía Blanca) and Universidad Nacional del Sur, Bahía Blanca, Buenos Aires, Argentina; g INRA-UMR 1095, Genetics, Diversity and Ecophysiology of Cereals, Institut National de la Recherche Agronomique-Université Blaise Pascal, Clermont-Ferrand, France; h Bayer Crop Science, 3500 Paramount Parkway Morrisville, NC 27560, USA; i Centre of the Region Haná for Biotechnological and Agricultural Research, Institute of Experimental Botany, Sokolovska 6, CZ-77200 Olomouc, Czech Republic; j Instituto de Recursos Biológicos, CIRN, INTA, Hurlingham, Buenos Aires, Argentina

**Keywords:** Bd, *Brachypodium distachyon*, CDS, coding DNA sequences, *dN*/*dS*, non-synonymous-to-synonymous substitutions, EST, expressed sequence tag, ISBP, insertion site-based polymorphism, IWGSC, International Wheat Genome Sequencing Consortium, LMP, long mate pair, MDA, multiple displacement amplification, NGS, next generation sequencing, Os, *Oryza sativa –* rice, Sb, *Sorghum bicolor –* sorghum, SE, single end, SSR, single sequence repeat, SNP, single nucleotide polymorphism, Chromosome 4D survey sequence, Gene annotation, Gene content, Synteny, Virtual gene order, *Triticum aestivum*

## Abstract

Survey sequencing of the bread wheat (*Triticum aestivum* L.) genome (AABBDD) has been approached through different strategies delivering important information. However, the current wheat sequence knowledge is not complete. The aim of our study is to provide different and complementary set of data for chromosome 4D. A survey sequence was obtained by pyrosequencing of flow-sorted 4DS (7.2×) and 4DL (4.1×) arms. Single ends (SE) and long mate pairs (LMP) reads were assembled into contigs (223 Mb) and scaffolds (65 Mb) that were aligned to *Aegilops tauschii* draft genome (DD), anchoring 34 Mb to chromosome 4. Scaffolds annotation rendered 822 gene models. A virtual gene order comprising 1973 wheat orthologous gene loci and 381 wheat gene models was built. This order was largely consistent with the scaffold order determined based on a published high density map from the *Ae. tauschii* chromosome 4, using bin-mapped 4D ESTs as a common reference. The virtual order showed a higher collinearity with homeologous 4B compared to 4A. Additionally, a virtual map was constructed and ∼5700 genes (∼2200 on 4DS and ∼3500 on 4DL) predicted. The sequence and virtual order obtained here using the 454 platform were compared with the Illumina one used by the IWGSC, giving complementary information.

## Highlights

• Survey sequence of *T. aestivum* chromosome 4D was obtained by pyrosequencing.
• Near 5700 genes were predicted on 4D chromosome, ∼2200 on 4DS and ∼3500 on 4DL.
• A 4D virtual gene order based on synteny with orthologous gene loci is proposed.
• Among group 4, higher collinearity exists between 4D and 4B as compared to 4A.
• Complementary data to that provided by IWGSC is presented, available at NCBI.

## 1 Introduction

Bread wheat (*Triticum aestivum* L.) is an essential component of the global food security mosaic, providing nearly one-fifth of the total calories of the world's population [1]. Since the mid-twentieth century, breeding has contributed to improve wheat crop production by increasing yield and the cultivated area along with the world's growth in population. However, recent studies indicate stagnation in wheat yields at a global level [2,3], which contrasts with the projected demands for agricultural crops expected to almost double by 2050 [4]. Therefore, raising the yield potential and stabilizing yields against the damaging effects of climate change are top priorities for agricultural science [2,5]. In this context, a better understanding of the wheat genome is central to unlock the full potential of natural genetic variation and to develop more effective breeding strategies for crop improvement.

Sequencing the bread wheat genome has been always a challenging task because of its size and complexity, resulting from its allohexaploid (2*n* = 6*x* = 42 AABBDD) nature and high content of repetitive DNA [6–8]. Bread wheat originated approximately 8000 years ago as a result of spontaneous interspecific hybridization (and subsequent chromosome duplication) between domesticated emmer *T. turgidum* ssp. *dicoccon* (2*n* = 4*x* = 28 AABB) and diploid *Aegilops tauschii* (2*n* = 2*x* = 14 DD) [9]. The combination of three large diploid genomes led the haploid complement of wheat genome to be as large as 17 Gb [10], making it about 40 times larger than the rice genome [11]. This magnitude reflects not only the sum of orthologous gene copies but also a high amount of repetitive sequence, which is estimated to represent 80% of the whole wheat genome [7,8].

Different approaches have been adopted to circumvent these restrictions and as a result, wheat genomics has moved forward steadily, although slower than that of other crops such as rice. Most of this progress has relied on comparative genomics among grasses and on the use of diploid progenitors to gain knowledge on bread wheat. The advent of high-throughput sequencing technologies – so-called next generation sequencing (NGS) – has a remarkable positive effect on sequencing capabilities, in terms of both speed and depth, at an economically accessible cost [12]. This has prompted the development of genome sequencing projects for a broad range of organisms, including first draft versions of several members of the Triticeae like bread wheat [13,14], the A genome donor *T. urartu* [15], and the D genome donor *Ae. tauschii* [16]. These studies provided valuable estimations of gene content, putative gene orders and genome organization. However, the preliminary status of such large and complex genomes may reveal only part of the entirety of genes present in wheat and, at the same time, the study of the A or D donor genomes may not reflect the current genome architecture of modern cultivated wheat due to reduction and rearrangements, for instance.

Among the wheat subgenomes, the D genome is characterized by its low level of genetic diversity, which has been an impediment to developing molecular tools for breeding programs, limiting the potential of introducing advantageous traits associated with this sub-genome. In particular, the low level of polymorphism presented by chromosome 4D has resulted in linkage maps with a few markers and the presence of large gaps (>30 cM) [17,18]. More recently, major advances in *Ae. tauschii* whole genome analysis have been reported, facilitating gene discovery and development of highly saturated genetic and physical maps [16,19]. These data, together with available NGS sequences of homoeologous wheat chromosomes 4A (IWGSC), 4B (IWGSC) and 4D (IWGSC, this study) represent valuable resources to get insights in the evolution of this set of homeologous chromosomes. In the same way, it will constitute an important resource to generate tools for gene discovery and breeding. Efforts to sequence and annotate chromosome 4D are therefore particularly relevant to obtain insights that will be key to develop genomics tools for wheat. In this study, we report the assembly and annotation of bread wheat chromosome arms 4DS and 4DL obtained by flow cytometry and multiple displacement amplification (MDA), combining 454 shotgun single end and LMP reads. It is expected that our approach will contribute to explore different regions of the 4D chromosome sequence and will make the 4D sequences available for wheat research community (JROL00000000).

## 2 Materials and methods

### 2.1 Plant material

Double ditelosomic stock 4D of bread wheat cv. Chinese Spring (2*n* = 40 + 2t4DS + 2t4DL) is a stable line in which chromosome 4D is maintained as a pair of telocentric chromosomes 4DS and 4DL [20]. Grains of this stock were kindly provided by Dr. Bikram Gill (Kansas State University, Manhattan, USA).

### 2.2 Chromosome sorting and DNA amplification

Aqueous suspensions of mitotic chromosomes were prepared from synchronized root tips as described by Vrána et al. [21]. The telosomes were sorted using a FACS Aria SORP flow cytometer (Becton Dickinson, San José, USA) into 40 μL sterile deionized water. Fluorescence *in situ* hybridization was used for purity assessment of sorted samples using probes for telomeric repeat, *Afa* and [GAA]_*n*_ repeats according to Kubaláková et al. [22]. Flow-sorted chromosomes were treated with proteinase and their DNA was extracted using a Microcon YM-100 column (Millipore Corporation, Bedford, USA), as described by Šimková et al. [23]. Chromosomal DNA was amplified by multiple displacement amplification (MDA), using an IllustraGenomiPhi V2 DNA amplification kit (GE Healthcare Bio-Sciences, Piscataway, USA).

### 2.3 Chromosome arm sequencing, assembly and validation

MDA-amplified DNA (500 ng) was used to prepare single end (SE) and 3-kb long mate pair (LMP) libraries using the GS FLX DNA library preparation kit, following the manufacturer's instructions (Roche Diagnostics). Libraries were sequenced across four and two full runs, respectively, using the Genome Sequencer FLX Instrument (Roche Applied Science, Penzberg, Germany) at Indear (CONICET, Rosario, Argentina).

*De novo* assembly was performed using the software Newbler 2.6 with default parameters. While other assembly software (including Mira, ABySS and WGS) were tested and analyzed, this dataset worked better with Newbler, as expected. The contig construction step was executed in single processor mode to avoid any possible problem created by the processing subdivision on low coverage sequencing. LMP reads were included in the contig step as single end reads to increase the coverage, obtaining a better representation of the chromosome content. Scaffolding was executed using Newbler 2.6 with default parameters.

Chromosome arm coverage was assessed using the Kmer Analysis Toolkit (KAT) (http://www.tgac.ac.uk/kat) by counting k-mer occurrence considering an index of 20-mer sized words representing the primer sequence of high confidence insertion site-based polymorphism (ISBP) markers, which represent unique single loci in the genome, thus revealing the underlying sampling distribution for unique motifs. ISBPs were designed using the ISBP Finder script [24] on preliminary SE contigs (data not shown).

In order to validate the LMP library efficiency, the read distance in the LMP was assessed by mapping paired reads against the SE contigs using the software Bowtie under default parameters [25]. Chromosome completeness was assessed by measuring the representation of wheat 4D chromosome bin-mapped ESTs [26], on both, assemblies and raw data using NUCmer [27] and kmer mapping approaches. On the other hand, chromosome 4D scaffolds and contigs were compared against the *Ae. tauschii* survey sequence genome and physical map, including SSR and SNP genetic maps [16,19]. Sequence comparisons were performed using the Mummer3 software package [27] with the following parameters: maxmatch −l 50 −g 50 −c 500. The datasets used in these comparisons are detailed in Table S1.

Supplementary Table S1 related to this article can be found, in the online version, at http://dx.doi.org/10.1016/j.plantsci.2014.12.004.

Supplementary Table S1 *Ae tauschii* and wheat datasets used to assess 4D scaffolds.

### 2.4 Gene prediction and annotation

The annotation of protein-coding genes with evidence-based quality indexing, and the identification of conserved non-coding sequences were performed using the structural and functional automatic annotation pipeline TriAnnot [28]. Given that the concept of the pipeline is the identification of full-length genes, only scaffolds of 6 kb or more were used to identify gene models. *In silico* gene predictions were combined with searches based on similarity using the algorithm BLAST [29] against full-length cDNAs, unigenes, and ESTs from Triticeae and other members of the Poaceae family, and the rice (*Oryza sativa*) proteome. Repeats and transposable elements were identified in wheat specific repeat databases, as described by Leroy et al. [28]. The gene models obtained from TriAnnot were classified into three categories: (1) high-confidence genes: full-length genes with well-established start and stop codons as well as exon-intron junction boundaries supported by biological evidences; (2) low-confidence genes: full-length genes in which start/stop codons and/or exon-intron junction boundaries are not well established; (3) putative pseudo-genes: predicted genes that would encode a sequence corresponding to <70% of a known protein.

The gene models predicted by the TriAnnot pipeline were functionally annotated as follows: BLASTX (*e*-value cut off ≤10e–10) searches were performed against the NCBI Viridiplantae protein database first. Then, the sequences with no hits were used to perform a BLASTX search against the NCBI nr protein database in order to make an assessment of the putative identities of the sequences. Annotation and mapping were done using the software BLAST2GO [30], which assigns Gene Ontology terms [31] (http://www.geneontology.org). GO annotation was completed by running the full suite of InterProScan under default parameters [32]. InterProScan combines different protein signature recognition methods native to the InterPro member databases into one resource that searches for the corresponding InterPro and GO annotations. Despite the fact that TriAnnot produces by itself a functional annotation [28], in this study we applied an in-house routine to produce an output compatible with ATGC, a web interface for the Chado database in use at the INTA annotation projects [33].

### 2.5 Construction of a syntenic build based on gene order in brachypodium, rice and sorghum

The GenomeZipper workflow [34] was applied with some adjustments. The comparison and integration of the 4DS and 4DL contigs into a linear reference gene order was achieved by exploiting the synteny with *Brachypodium distachyon* (Bd), *Oryza sativa* (Os) (rice), and *Sorghum bicolor* (Sb) (sorghum) sequenced genomes.

Repetitive DNA was identified using Vmatch (http://www.vmatch.de) against the MIPS-REdatPoaceae v9 repeat library. The MIPS repeat library contains known grass transposons from the Triticeae Repeat Database, as well as *de novo* detected LTR retrotransposon sequences from several grass species. Vmatch was run with the following parameters: 70% identity cut-off, 100 base pairs minimal length, seed length 14, exdrop 5 and e-value 0.001 and each identified repeat was masked by “N's”. Only sequences with a minimum of 100 base pairs non-masked nucleotides were considered for the subsequent steps.

All the repeat-free 454 contigs from 4DS and 4DL were compared to *Brachypodium*, rice and sorghum proteins using BLASTX. As stringent filtering criteria (a) only the first best hit with, (b) a minimal alignment length of 30 amino acids and (c) a minimal sequence identity of 75% (*Brachypodium*)/70% (rice, sorghum) was used in each case. In-house software called ChromoWIZ was used to calculate the degree of synteny between the above-mentioned datasets. ChromoWIZ is based on a sliding window approach. For each genetic position (window 0.5 Mb, window shift 0.1 Mb) the number of conserved genes was calculated and heat maps were constructed. The heat maps showed the high conserved (syntenic) regions using a blue-red colored map.

### 2.6 Setting the virtual gene order

A genetic map of *T. aestivum* chromosome 4D based on SNPs [35], was used as a backbone to order and position the contigs along with wheat ESTs from the HarvEST database (http://harvest.ucr.edu), wheat full-length cDNAs from NCBI and TriFLDB [36] (http://trifldb.psc.riken.jp/v3/download.pl) and 822 4D wheat gene models predicted by the TriAnnot pipeline (this study). The orthologous genes were anchored to the backbone *via* bi-directional BLAST hits. The genes that gave positive matches but were not associated to any “marker” were located according to their position in the corresponding reference genome.

The virtual gene order established using GenomeZipper was compared with the scaffold order obtained using *Ae. tauschii* genetic maps [16]. All comparisons were visualized using the software Circos [37] (http://www.circos.ca/). 4D bin-mapped ESTs [26] with positive BLAST hits against contigs carrying syntenic genes and scaffolds anchored by *Ae. tauschii* SSRs as well as coding DNA sequences (CDSs) and repetitive sequences from scaffolds predicted by TriAnnot (see below), were included in this analysis.

Additionally, chromosome 4D gene content obtained from GenomeZipper with 454 data was compared with the 4D GenomeZipper obtained with the Illumina platform by the IWGSC [14]. Therefore, the number of shared and specific loci from both gene maps was determined.

### 2.7 Assessing the number of genes

An estimation of the total number of genes present on chromosome 4D was performed by adding to the syntenic genes obtained with GenomeZipper the predicted proportions of uncovered and non-syntenic genes obtained from the current assembly. In order to do this, the first step was to estimate the gene coverage (%) of the contigs using a subset of 550 wheat ESTs bin-mapped on the 4D chromosome [26]. This was done using the algorithm BLASTN (>95% of identity and a minimum length of 100 nt). The second step was identifying which of the ESTs that were positively matched in step one, were present in contigs included in the virtual map constructed by GenomeZipper. As this virtual map included only those contigs with regions syntenic to other grass genomes, the percentage of positive and negative ESTs matches were used as an estimation of the percentage of syntenic and non-syntenic gene loci putatively present on the 4D chromosome.

### 2.8 Comparative analysis against wheat 4A and 4B chromosomes

The virtually ordered gene maps generated by GenomeZipper were used to perform comparative analyses of the syntenic conserved gene space among wheat chromosomes of the homeologous group 4, 4D (454 platform), 4B and 4A (Illumina platform, IWGSC) [14]. All conserved orthologous genes, represented by common anchored *Brachypodium*, rice and sorghum genes in the linear gene maps, were identified and their positions on the individual wheat chromosome arms were visualized by connecting lines.

### 2.9 Rates of coding sequence evolution

The gene models predicted by the TriAnnot pipeline were used to create a set of orthologs using the orthoMCL software version 2.0.9 [38] under default parameters. We compared wheat 4D predicted proteins, *B. distachyon* and *Ae. tauschii* proteomes as follows: the protein sets were pairwise aligned using ClustalW2 program version 2.1 [39] and the CDSs were translated and aligned using tranalign tool from the EMBOSS package version 6.3.1 [40] to generate alignments of nucleic sequences from aligned proteins. Alignments consisting of more than 20% gaps were removed.

To estimate the rate of coding sequence evolution, the non-synonymous to synonymous substitution ratios (*dN*/*dS* ratio) were calculated for all possible comparisons using the method of Yang and Nielsen [41] as implemented in the PAML package (estimating *dN*/*dS* ratios).

## 3 Results

### 3.1 DNA preparation and 454 sequencing

**Fig. 1:**
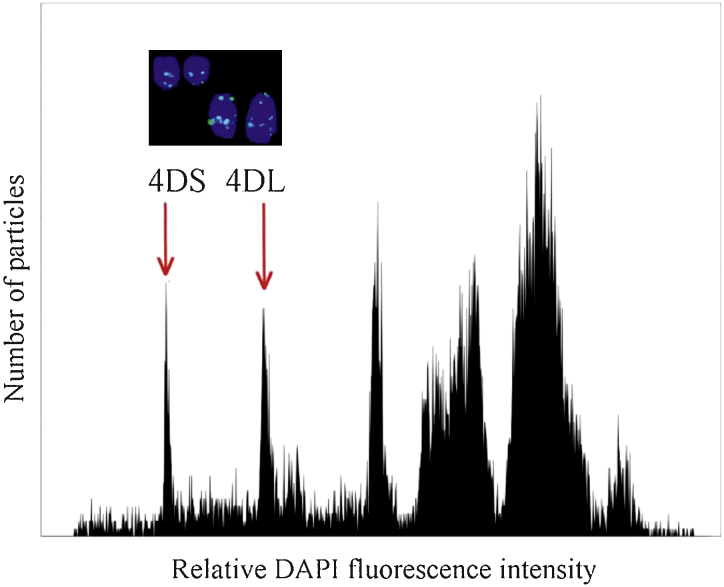
Histogram of relative DAPI fluorescence (flow karyotype). Histogram obtained after flow cytometric analysis of mitotic metaphase chromosomes of double ditelosomic line 4D of bread wheat cv. Chinese Spring. Peaks corresponding to telosomes 4DS and 4DL are well discriminated, which facilitated their flow sorting. Sorted chromosome arms were identified after FISH with probes for Afa repeat (yellow-green), which results in characteristic banding pattern (inset). *X*-axis: relative DAPI fluorescence intensity; *Y*-axis: number of particles.

Flow cytometric analysis of DAPI-stained chromosomes prepared from double ditelosomic line 4D of cv. Chinese Spring resulted in a histogram of fluorescence intensity (flow karyotype) on which the 4DS and 4DL telosomes formed distinct peaks (Fig. 1). This enabled sorting 4DS and 4DL arms with the average purities of 91.42% and 86.63%, respectively. The sorted fractions were contaminated by a random mixture of fragments of various chromosomes and chromatids without prevalence of a specific chromosome. A total of 106,000 and 59,000 of 4DS and 4DL arms, respectively were sorted and used for DNA amplification. Two separate DNA bulks from flow-sorted arms were prepared using multiple displacement amplification (MDA). To limit representation bias, each DNA bulk was pooled from several independent MDA reactions. The 4DS bulk was combined from three amplifications and yielded 9.63 μg DNA. Similarly, the 4DL bulk yielded 5.44 μg DNA and contained DNA of four MDA reactions. MDA-amplified DNA yielded short fragments, suitable for the construction of a 3 kb-LMP library.

### 3.2 Sequence assembly and validation

**Supplementary Fig. S1:**
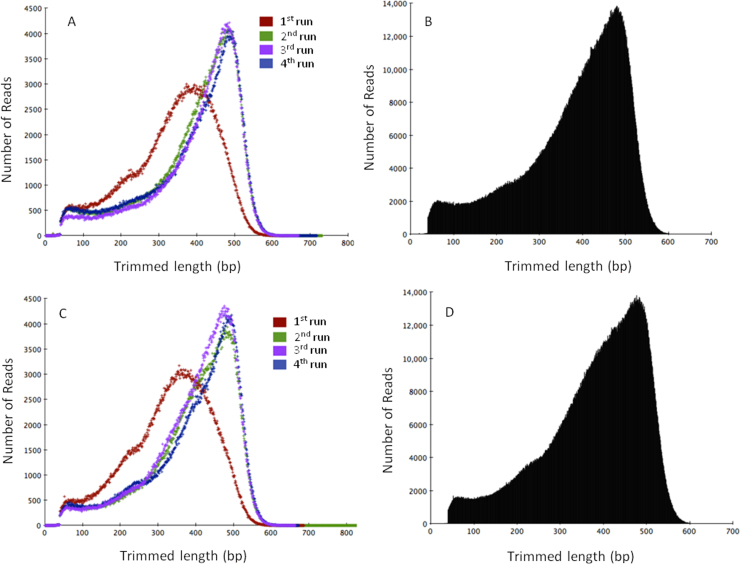
Single end read length histograms for trimmed reads of *T. aestivum* chromosome 4D. (A) 4D short arm chromosome individual run profiles; (B) 4D short arm chromosome whole read data set; (C) 4D long arm chromosome individual run profiles; (D) 4D long arm chromosome whole read data set.

**Supplementary Fig. S2:**
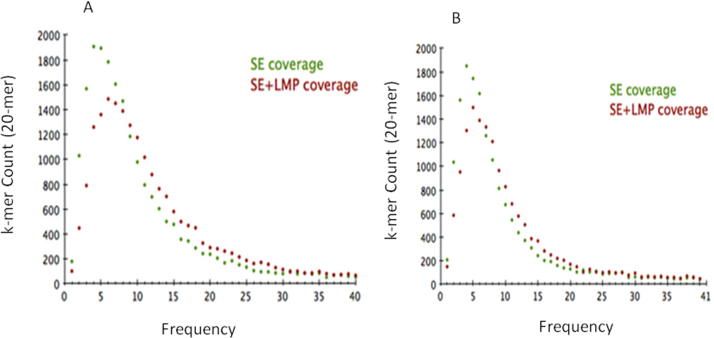
*T. aestivum* 4D chromosome arms sequence depth estimation using K-mer spectra plots [81]. (A) Comparison of short arm coverage given by single reads (green) vs. single read plus long mate pair reads (red); (B) comparison of long arm coverage given by single reads (green) vs. single read plus long mate pair reads (red).

Sequencing yields, read average size and coverage generated by the 454 runs are shown in Table S2 and Fig. S1. Total coverage including SE and LMP reads, considering the arm sizes estimated by Šafář et al. [42] was 7.2× for the short arm and 4.1× for the long arm. These calculations were confirmed by kmer spectra plots (http://www.tgac.ac.uk/kat) presented in Fig. S2.

Supplementary figures and table related to this article can be found, in the online version, at http://dx.doi.org/10.1016/j.plantsci.2014.12.004.

Supplementary Table S2 Statistics of single end (SE) and long mate pair (LMP) reads used in this study.

**Supplementary Fig. S3:**
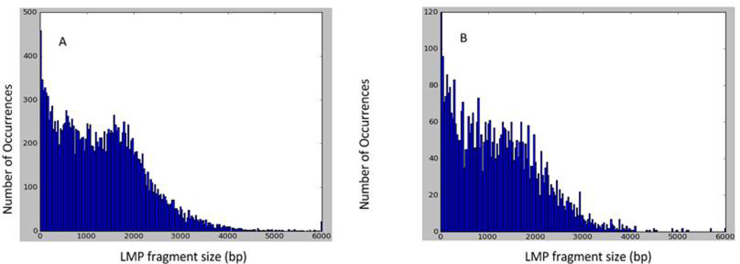
Long mate pair (LMP) fragment length distribution assessed on a subset of *T. aestivum* 4D contigs ≥5 kb. (A) 4D short arm chromosome LMP fragment distribution. (B) 4D long arm chromosome LMP fragment distribution.

**Supplementary Fig. S4:**
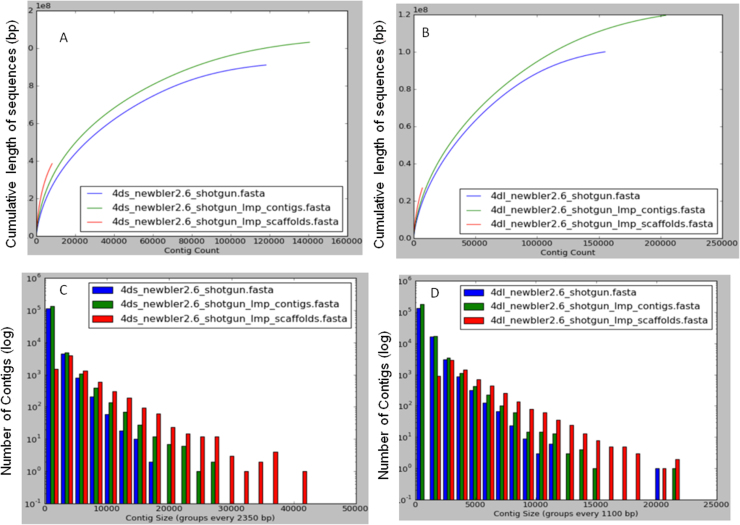
Comparison of 4D chromosome arm assembly generated by Newbler using single end reads to generate contigs (blue), Single end reads plus long mate pair reads as single end reads to generate contigs (green) and all single end reads plus long mate pair reads to generate scaffolds (red). The contigs/scaffolds are sorted in descending order of sizes. (A) Cumulative length plots of chromosome 4D short arm assembly; (B) cumulative length plots of chromosome 4D long arm assembly; (C) histogram plot of contig sizes for 4D short arm assembly; (D) histogram plot of contig sizes for 4D long arm assembly.

LMP end distance evaluation, conducted *a posteriori* on the assembled content, showed a sub-optimal fragment size distribution around the expected 3-kb insert size, with the prevalence of short fragments and a slight peak at approximately 1.6 kb (Fig. S3). Contig assemblies yielded 103 and 120 Mb for chromosome arms 4DS and 4DL, respectively, with a repeat content estimated in 67 and 70% (Table 1). Scaffolding was performed with additional 3-kb LMP reads yielding 38 and 27 Mb of 4DS and 4DL assembly, respectively. Statistics for the assemblies are shown in Table 1. Cumulative data obtained from contig and scaffold assemblies was lower than 142.1 Mb (L50 3,278 bp) and 347.6 Mb (L50 1013 bp) from the same chromosome using the Illumina platform [14]. However, the scaffold assemblies obtained in this study using the 454 platform showed superior L50 contig lengths, estimated in 5517 bp (4DS) and 3998 bp (4DL) showing an advantage of this technology, that provides longer reads than Illumina one. Contributions of LMP read on SE contigs and scaffold assemblies are shown as cumulative length plots (Fig. S4A and B) and contig/scaffold size histograms (Fig. S4C and D).

Supplementary figures related to this article can be found, in the online version, at http://dx.doi.org/10.1016/j.plantsci.2014.12.004.

**Supplementary Fig. S5:**
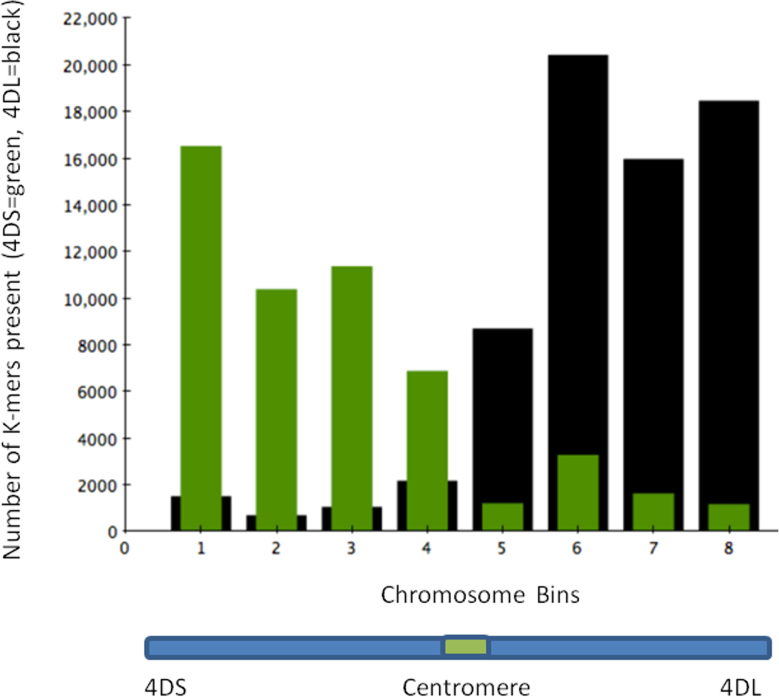
Assembly validation using 4D bin-mapped ESTs. Green bars represent 4D short arm binned EST k-mers present in the 454 short and long arm assembly (all contigs), respectively. Black bars represent 4D long arm binned EST k-mers present in the 454 long and short arm assembly (all contigs), respectively. Legend of Bins: Bin1: 4DS2-0.82–1.00, Bin2: 4DS3-0.67–0.82, Bin3: 4DS1-0.53–0.67, Bin4: 4DS1-0.53, Bin5: 4DL9-0.31, Bin6: 4DL9-0.31–0.56, Bin7: 4DL13-0.56–0.71, Bin8: 4DL12-0.71–1.00.

Chromosome completeness evaluation based on the presence of k-mers from 4D bin-mapped wheat EST sequences [26], reveals that approximately 62.5% of the expected ESTs are present in the assembly out of 66.5% present in the reads. This assessment revealed an overall better assembly for the short arm, showing a slight increase in 4× EST kmer, which is consistent with the higher coverage and cleaner content distribution (Fig. S5). In addition, the occurrence of a low amount of kmer from bin-mapped ESTs in the opposite chromosome arm indicated a low level of cross-contamination in the flow-sorted input DNA samples (Fig. S5).

Supplementary Fig. S5 related to this article can be found, in the online version, at http://dx.doi.org/10.1016/j.plantsci.2014.12.004.

**Supplementary Fig. S6:**
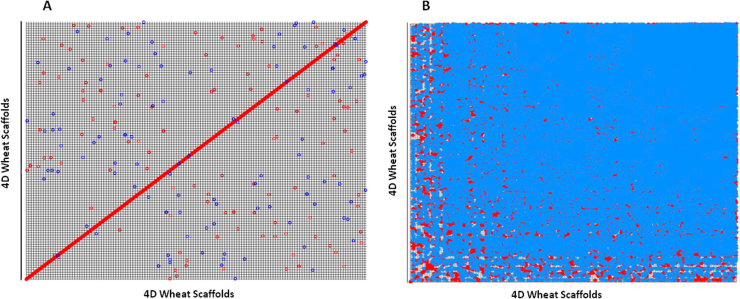
Self-comparison of *T. aestivum* 4D scaffolds. Shown are the results obtained using exact matches of (A) 50 nucleotides and (B) 20 nucleotides, with a cluster size of 500 nucleotides.

Wheat chromosome 4D arm scaffold assemblies were contrasted against *Ae. tauschii* genome datasets [16,19], as a complementary strategy for assessing chromosome completeness under the assumption that these are related genomes with a high level of sequences conservation. Due to the high amount of repetitive elements in wheat/*Ae.tauschii* sequence comparisons, the likelihood of misleading results was minimized by using the Mummer software [43] with a conservative approach defined by self-comparison of the 4D scaffold sequences (Fig. S6). In this way, 4D scaffolds were observed as non-repetitive and therefore redundant matches were avoided. Using this approach, the 65 Mb wheat 4D assembly was aligned to: (1) 180 Mb *Ae. tauschii* chromosome 4 anchored scaffolds [16]; and (2) ∼7 Mb *Ae. tauschii* chromosome 4 extended markers [19]. These comparisons yielded 7410 (49%) 4D scaffolds that matched *Ae. tauschii* chromosome 4 sequences. The remaining 7808 unmatched 4D scaffolds were challenged against the *Ae. tauschii* whole-genome scaffolds larger than 1 kb, comprising 4.05 Gb of sequence. By this approach 7700 4D scaffolds showed positive matches with the *Ae. tauschii* genome. 2133 (14%) 4D scaffolds rendered positive matches to *Ae. tauschii* scaffolds anchored to chromosomes other than 4, including 1D (281 scaffolds), 2D (338 scaffolds), 3D (357 scaffolds), 5D (602 scaffolds), 6D (278 scaffolds) and 7D (277 scaffolds). This 14% of inferred contamination is consistent with the observed purity of sorted chromosomes (91.42% and 86.63% for 4DS and 4DL, respectively). It is interesting to note that 5567 (37%) scaffolds matched to unanchored *Ae. tauschii* scaffolds. Several of these sequences probably belong to chromosome 4 but could not be assigned to a particular chromosome in the whole genome sequencing approach followed previously by Jia et al. [16]. The remaining 108 scaffolds (1%) showed similarity to bacterial genes or unknown sequences, as revealed by BLASTN searches against nucleotide databases. These could come from organelle DNA or simply contamination and were excluded from the 4D sequences.

Supplementary Fig. S6 related to this article can be found, in the online version, at http://dx.doi.org/10.1016/j.plantsci.2014.12.004.

### 3.3 Gene prediction and annotation

**Supplementary Fig. S7:**
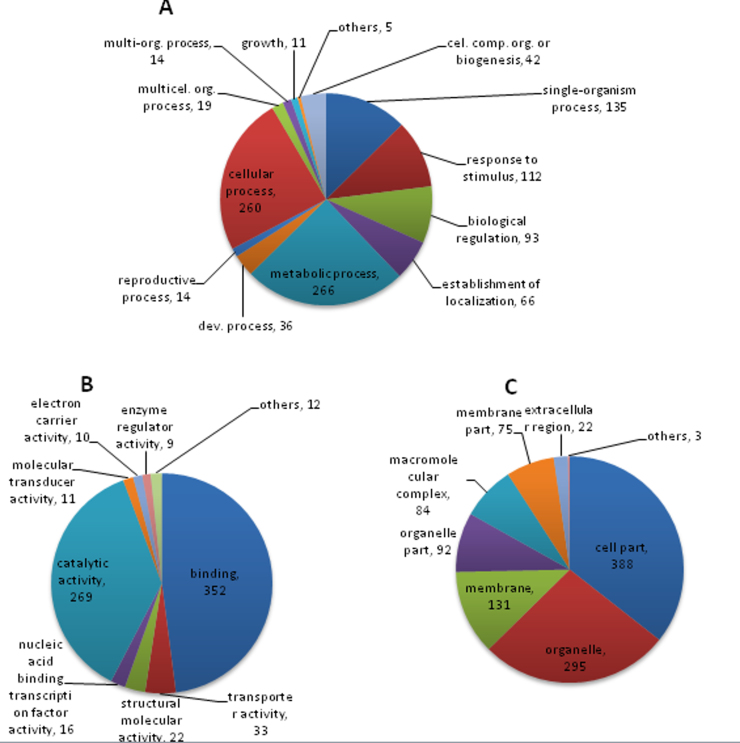
Functional annotation of gene model sequences from *T. aestivum* 4D chromosome. GO terms were assigned onto gene model sequences identified by the TriAnnot pipeline from 4DS and 4DL scaffolds. Pie charts were constructed showing the most abundant GO term assigned for each sequence in three categories: (A) Biological process; (B) molecular function and; (C) cellular component. The number of different sequences annotated in each term is also indicated.

Using the TriAnnot pipeline, 2611 scaffolds larger than 6 kb were analyzed and a total of 822 gene models were identified (595 on 4DS and 227 on 4DL). This includes 100 high-confidence genes, 480 low-confidence genes and 242 putative pseudogenes (hit coverage <70%) (Table 2). Differences in gene number per chromosome arm can be explained by the differences in the sequence coverage. From 822 gene models, 614 were functionally annotated by one or more GO terms. The distribution of GO term annotations is summarized in Fig. S7.

On the other hand, among all of the scaffold sequences,1073 were assigned to biological process terms, with the most frequent being metabolic (24.8%), cellular (24.2%) and single-organism process (12.6%; mostly protein and nucleic acid metabolism; Fig. S7A). Molecular function terms were assigned to 734 scaffold sequences, with binding (48%; mostly nucleic acid binding) and catalytic activity (37%; hydrolase and transferase activity) as predominant terms (Fig. S7B). In the case of cellular component, to which 1090 scaffold sequences were assigned, cell part (35.6%) and organelle (27.1%) were the most abundant terms-mostly proteins associated with plastid, cytoplasmic membrane-bounded vesicle, mitochondrion and nucleus (Fig. S7C). The relevance of GO terms like protein and nucleic acid metabolism, binding, and catalytic activity, as well as plastid and mitochondrion observed in our sample of 4D gene models are in agreement with the GO terms observed for chromosome 5A and chromosome arm 1AL [44,45]. Details of GO results and non-coding annotation are available online (http://atgc-wheat4D.inta.gob.ar).

Supplementary Fig. S7 related to this article can be found, in the online version, at http://dx.doi.org/10.1016/j.plantsci.2014.12.004.

### 3.4 Construction of a linear ordered virtual gene map (GenomeZipper)

**Fig. 2:**
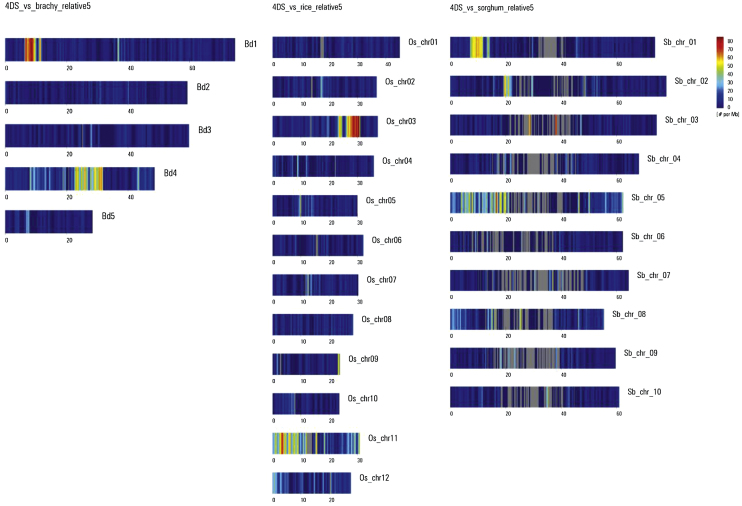
GenomeZipper analysis of wheat chromosome 4DS. Wheat 4DS contigs mapped onto the genomes of *Brachypodium distachyon* (A) *Oryza sativa* (B) and *Sorghum bicolor* (C) using the GenomeZipper approach. The heat map depicts read density across each of the chromosomes, with a blue-red color scale (blue = 0, red = 80).

**Fig. 3:**
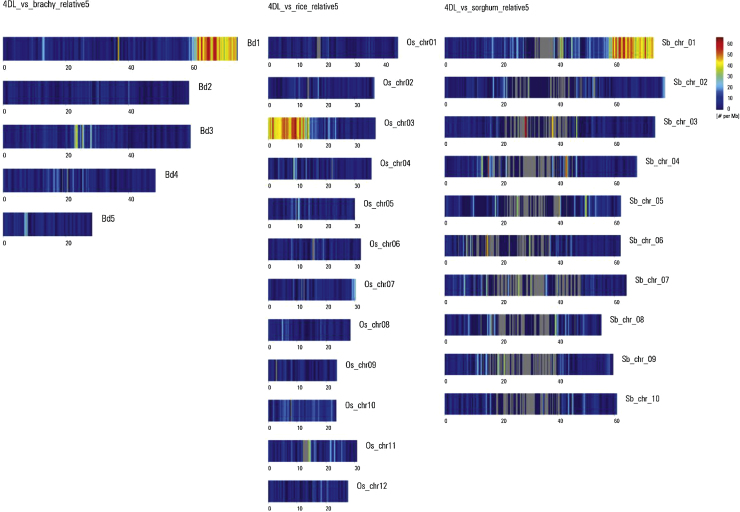
GenomeZipper analysis of wheat chromosome 4DL. Wheat 4DL contigs mapped onto the genomes of *Brachypodium distachyon* (A) *Oryza sativa* (B) and *Sorghum bicolor* (C) using the GenomeZipper approach. The heat map depicts read density across each of the chromosomes, with a blue-red color scale (blue = 0, red = 60).

Using a series of bioinformatics tools (ChromoWiz, GenomeZipper) that integrated 4D contigs with the reference genomes of rice (*Oryza sativa* – Os), sorghum (*Sorghum bicolor* – Sb), and *B. distachyon* (Bd), 1973 genes from chromosome 4D were ordered as syntenic blocks. Blocks of conserved synteny were observed on Bd chromosomes 1 and 4, Os chromosomes 3, 9 and 11 and Sb chromosomes 1, 2 and 5 for the 4DS chromosomal arm (Fig. 2), and on Bd chromosome 1, Os chromosome 3 and Sb chromosome 1 for the 4DL chromosome arm (Fig. 3).

**Supplementary Fig. S8:**
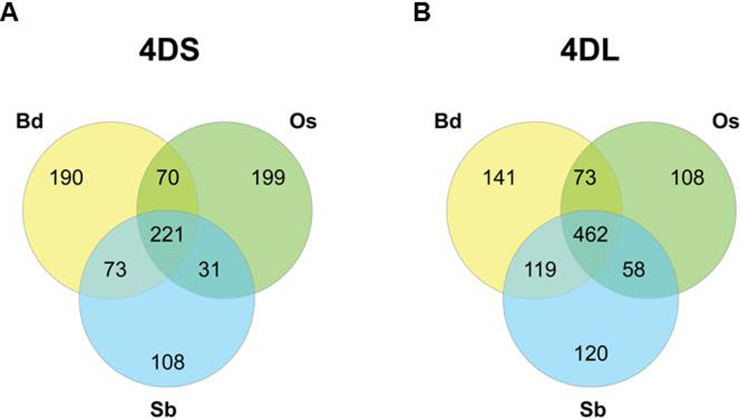
Venn diagram displaying the number of genes from *T. aestivum* 4D chromosome supported by other reference genomes. (A) 4DS genes supported by one (497 genes = 55.7%), two (174 genes = 19.5%) or three (221 genes = 24.8%) reference genomes. (B) 4DL genes supported by one (369 genes = 34.1%), two (250 genes = 23.2%) or three (462 genes = 42.7%) reference genomes. Bd: *Brachypodium distachyon*, Os: *Oryza sativa* (rice), Sb: *Sorghum bicolor* (sorghum).

A total of 892 syntenic genes were identified on chromosome 4DS supported by three (24.8%), two (19.5%) or one (55.7%) of the three reference organisms (Fig. S8). For chromosome 4DL, the distribution of the 1081 genes was supported by three (42.7%), two (23.2%) or one (34.1%) of the reference genomes (Fig. S8). Interestingly, 42.7% of the 4DL genes were supported by the three reference genomes. On the other hand only 24.8% of the 4DS genes were supported by all three genomes and the majority (55.7%) was supported by only one reference genome. This is suggestive of a higher frequency of gene loss in the 4DS collinear regions after divergence of these reference genomes, compared to the 4DL collinear regions.

Supplementary Fig. S8 related to this article can be found, in the online version, at http://dx.doi.org/10.1016/j.plantsci.2014.12.004.

GenomeZipper was further used to determine the structure and order of gene loci identified on wheat 4DS and 4DL contigs based on collinearity to the reference grass genomes, using the public SNP map from *T. aestivum* as a marker backbone [35]. All syntenic genes associated with SNP markers (based on the best bidirectional BLAST hit) were anchored to the backbone and additionally tagged genes without marker associations were ordered following the concept of synteny and closest evolutionary distance. The 4DS and 4DL GenomeZipper-based tentative gene order including associated information is provided as a Supplemental Excel file (Table S3).

Supplementary Table S3 related to this article can be found, in the online version, at http://dx.doi.org/10.1016/j.plantsci.2014.12.004.

Supplementary Table S3 4DS and 4DL Genome Zipper-based gene orders.

Wheat ESTs obtained from the HarvEST database (http://harvest.ucr.edu) wheat full-length cDNA orthologs to Bd, Os and Sb, and gene models predicted by TriAnnot (270 (45.4%) for 4DS and 111 (48.9%) for 4DL) were also integrated on the virtually ordered linear map.

To obtain a putative linear gene order for the entire wheat chromosome 4D, the gene maps of 4DS and 4DL were combined in one non-redundant map. By combining the two gene maps, the centromere region was revealed to be at 26.2 cM. This backbone included 16 SNPs on 4DS (–26.2 cM, clustered in 7 blocks) and also 16 SNPs on 4DL (27.21–98.81 cM, clustered in 7 blocks). Thus, considering the markers anchored by the different strategies used, the total number of loci that could be virtually mapped on the 4D chromosome was 902 and 1092 for 4DS and 4DL, respectively (Table 3).

### 3.5 Gene content

The virtual map described above includes a number of loci and can be viewed as a sub-sample of the total gene content present in chromosome 4D. We considered that coverage and non-syntenic sequences should be taken into account to obtain a better approximation of 4D gene content.

In order to overcome the bias introduced by the coverage, a BLASTN analysis of 4D contigs against a subset of 550 non-redundant EST loci, unambiguously bin-mapped on chromosome 4D [26], was performed. This analysis showed rates of matches of 67.8% (143/211 ESTs) for 4DS and 59.3% (201/339 ESTs) for 4DL. These values were used as an estimation of gene coverage of the assembly. As a result, under a theoretical coverage condition of 100%, the syntenic genes detected by GenomeZipper would increase from 892 to 1316 genes (4DS) and from 1081 to 1823 genes (4DL). This would yield a total of 3139 syntenic gene loci for chromosome 4D.

By contrast, to estimate the proportion of non-syntenic genes present in our contigs, those contigs that matched the 344 ESTs were BLAST searched against the 4D contigs that were included into the GenomeZipper virtual map. This analysis yielded 192 syntenic (60.1% 4DS and 52.4% 4DL) and 152 non-syntenic ESTs (39.9% 4DS and 47.3% 4DL). If these percentages of syntenic and non-syntenic genes were uniform all along chromosome 4D, another 2510 non-syntenic genes should be added to this chromosome, giving a final number of 5649 estimated genes.

### 3.6 Comparative analyses

#### 3.6.1 4D GenomeZipper *vs Ae. tauschii* genetic map and 4D deletion map

The virtually ordered linear map constructed using GenomeZipper was first contrasted against the 4D scaffolds ordered based on the genetic map of the ancestral D genome donor, *Ae. tauschii* [16].

**Fig. 4:**
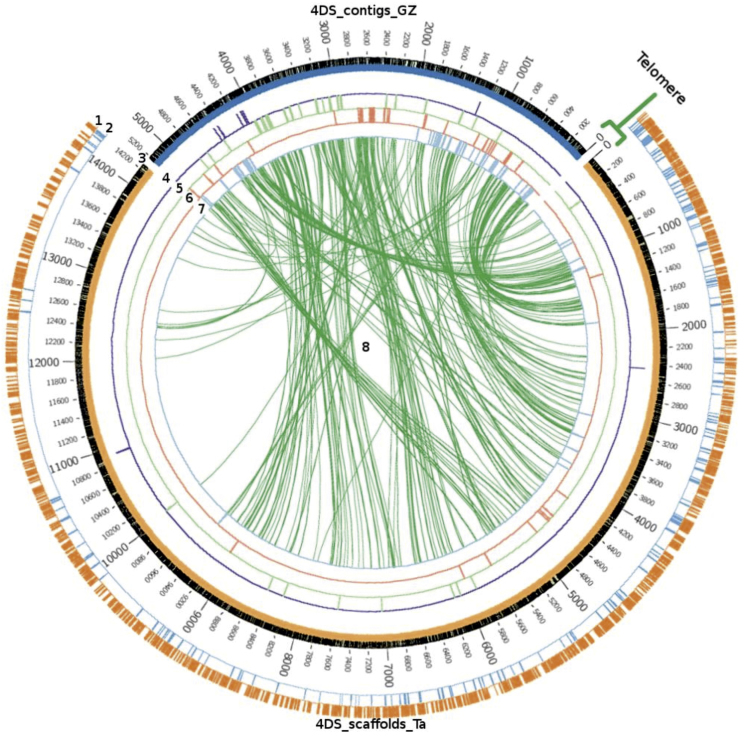
4DS GenomeZipper *vs. Ae. tauschii* genetic map and 4D deletion map. Comparison of 4DS scaffolds anchored and ordered according to the *Ae. tauschii* genetic map from Jia et al. [16] and the syntenic ordering of contigs obtained by GenomeZipper (this work). Ring 1, annotated repetitive regions from scaffolds. Ring 2, annotated genesfrom scaffolds. Ring 3, 4DS scaffolds (orange) and Genome Zipper contigs (blue) ordered in scale, where 0 represents the telomeres. Ring 4, (blue) ESTs mapped on C-4DS1-0.53 deletion bin. Ring 5 (green) ESTs mapped on 4DS1-0.53–0.67. Ring 6, (red) ESTs on 4DS3-0.67–0.82. Ring 7, (blue) ESTs on 4DS2-0.82–1.00. Ring 8, (green lines) matches between contigs ordered by GenomeZipper and scaffolds anchored to the *Ae. tauschii* genetic map. EST mapping data from deletion bins was obtained from Miftahudin et al. [26].

For 4DS, well-preserved collinearity was observed in both telomeric and pericentromeric regions. However, some inconsistencies in the gene order were observed in distal parts of the pericentromeric region (Fig. 4, ring 8). It should be noted that most of the wheat scaffolds ordered according to the *Ae. tauschii* map and anchored in the centromeric region showed no matches with contigs carrying syntenic genes from the 4DS GenomeZipper map, suggesting a very limited number of syntenic genes within this chromosomal region (Fig. 4, ring 8).

A second comparison based on the deletion map of 4DS [26] also showed a relatively lower representation of bin-mapped ESTs on the C-4DS1-0.53 deletion line (centromere, Fig. 4, ring 4) related to ESTs bin-mapped in chromosome regions defined by deletion lines 4DS1-0.53–0.67 (ring 5), 4DS3-0.67–0.82 (ring 6) and 4DS2-0.82–1.00 (ring 7). Moreover, the order of bin-mapped ESTs on the C-4DS1-0.53 and 4DS1-0.53–0.67 (ring 5) deletion lines mostly agreed with the order of the contigs obtained by GenomeZipper. A partial overlap observed between the ESTs bin mapped on the 4DS3-0.67–0.82 and 4DS2-0.82–1.00 deletion lines (rings 6 and 7) indicated some inconsistency between physical and syntenic data. Additionally, and in agreement with 4D scaffold order based on the *Ae.tauschii* map, a subset of ESTs that mapped on the telomeric bin 4DS2-0.82–1.00 matched with the GenomeZipper centromeric region (ring 7).

**Fig. 5:**
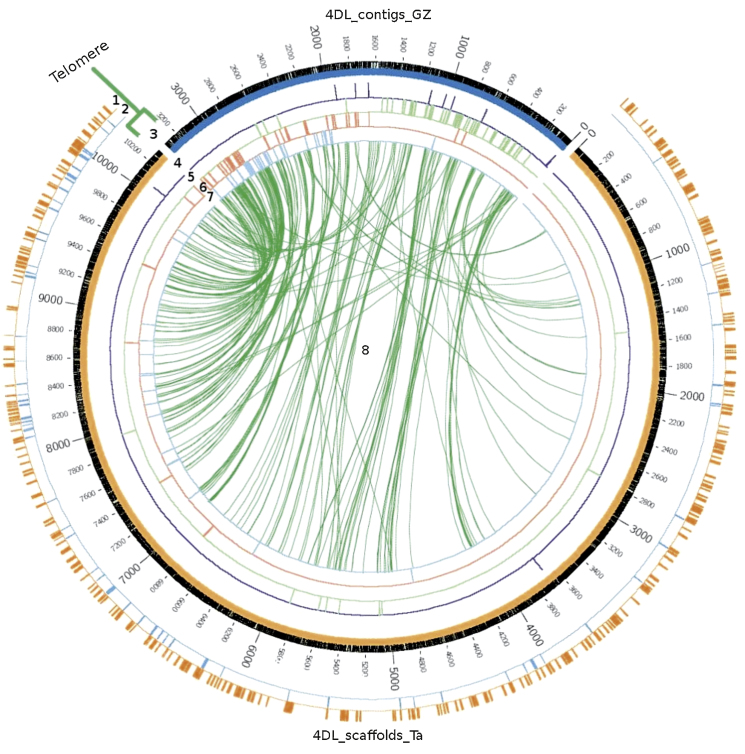
4DL GenomeZipper *vs. Ae. tauschii* genetic map and 4D deletion *map.* Comparison of 4DL scaffolds anchored and ordered according to the genetic map from Jia et al. [16] and the syntenic ordering of contigs obtained by the GenomeZipper approach (this work). Ring 1, annotated repetitive regions from scaffolds. Ring 2, annotated genes from scaffolds. Ring 3, 4DL scaffolds (orange) and GenomeZipper contigs (blue) ordered in scale where 0 represents the centromeres. Ring 4, (blue) ESTs mapped on C-4DL9-0.31 deletion bin. Ring 5, (green) ESTs mapped on 4DL9-0.31–0.56. Ring 6, (red) ESTs in 4DL13-0.56–0.71. Ring 7 (blue) ESTs in 4DL12-0.71–1.00. Ring 8, (green lines) matches between contigs ordered by GenomeZipper and scaffolds anchored to the *Ae. tauschii* genetic map. Deletion bin-mapping data of ESTs was obtained from Miftahudin et al. [26].

A similar analysis was performed with the 4DL GenomeZipper map. Well-preserved collinearity was again observed in the pericentromeric and proximal telomeric regions with a few inconsistencies in the distal telomeric region (Fig. 5 ring 8). Also as in 4DS but to a lesser degree, wheat scaffolds ordered according to the *Ae. tauschii* map and anchored in the centromeric region showed a low number of matches with contigs carrying syntenic genes from the 4DL GenomeZipper map.

However, plotting bin-mapped ESTs on C-4DL9-0.31 (ring 4) and 4DL9-0.31-0.56 (ring 5) lines revealed an overlapping pattern at proximal centromeric regions. The ordering of the bin-mapped ESTs from 4DL13-0.56–0.71 (ring 6), was bimodal, with a first cluster of markers on the proximal centromeric region and a second cluster on the distal centromeric region partially overlapping with markers bin-mapped on the line 4DL12-0.71–1.00 (ring 7). As in 4DS, ESTs bin-mapped at the centromere region were less represented than ESTs bin-mapped in other chromosomal regions.

An even distribution of repetitive elements annotated using the TriAnnot pipeline (mainly transposable elements) was observed on the 4D scaffolds from both chromosome arms (Figs. 4 and 5, ring 1). The identification and characterization of the repetitive sequences of chromosome 4D are being reported by Garbus et al. [46]. Contrastingly, genes showed a different distribution pattern characterized by more dense distribution in telomeric regions (Figs. 4 and 5, ring 2).

#### 3.6.2 Wheat group 4 interchromosomal comparison

The syntenic conserved gene space of wheat chromosome 4D was compared against the syntenic conserved regions in wheat chromosomes 4A and 4B [14]. Whereas the two wheat chromosomes 4D and 4B are entirely collinear except for some rearrangements, the collinearity with chromosome 4A is more fragmented. Due to the presence of an ancestral pericentric inversion that includes almost all chromosome 4A [47], most of the long arm of chromosome 4A shows conserved synteny to the short chromosome arm of 4D, whereas the short arm of 4A and a small part of 4AL are collinear with the long arm of 4D. The detection of the 4A pericentric inversion by the Genome Zipper approach reinforces the applicability of this approach to define virtual gene orders in grasses.

**Supplementary Fig. S9:**
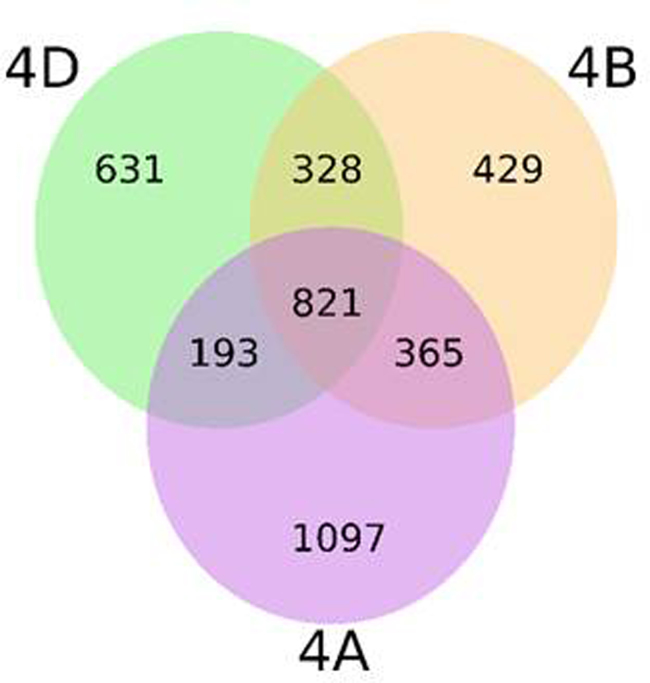
Venn diagram displaying the number of syntenic genes shared by wheat chromosomes 4A/4B, 4A/4D and 4B/4D and the three ways intersect.

**Supplementary Fig. S10:**
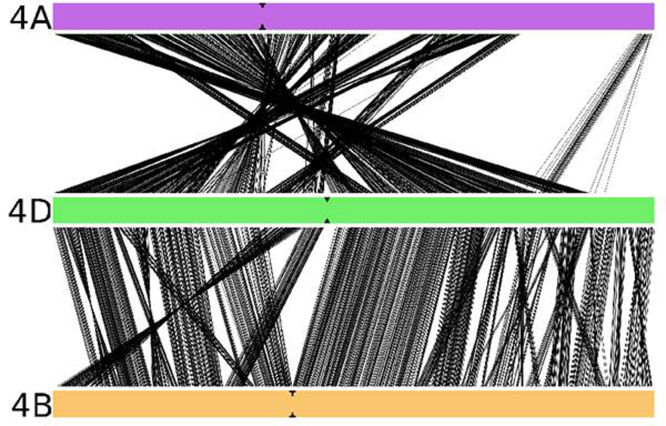
Gene loci shared among entire chromosomes of *T. aestivum* homeologous group 4. The putatively orthologous genes between the three wheat chromosomes are connected with lines.

4D/4A and 4D/4B comparisons were supported by 1014 and 1149 syntenic conserved gene loci, respectively (Fig. S9). The lower number of conserved syntenic gene loci observed between 4D/4A, in addition to a higher number of gene loci in 4A could be accounted by the 5AL and 7BS translocations present in chromosome 4A [26,47–49] and not present in chromosomes 4D and 4B. The structure of wheat chromosome 4A, as inferred by Devos et al. [47] and Miftahudin et al. [26], coincides with the structure of 4A except for the position of a short segment on the long chromosome arm (C region in Hernandez et al. [48]. In a previous study, this segment was positioned after the segment involved in the pericentric inversion and before the segments involved in the interchromosomal translocation with chromosomes 5AL and 7BS. Our 4D – 4A comparison revealed that the segment mentioned above is positioned at the terminal end of the long arm of chromosome 4A. In addition, virtual rearrangements can be observed in both comparisons, which disturb the ordering in small regions along the chromosomes (Fig. S10).

Supplementary figures related to this article can be found, in the online version, at http://dx.doi.org/10.1016/j.plantsci.2014.12.004.

### 3.7 Rates of coding sequence evolution

**Fig. 6:**
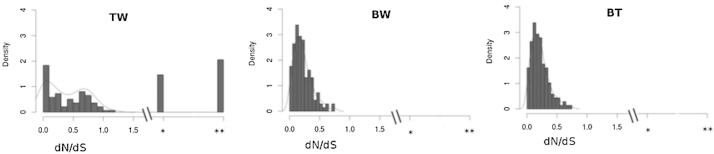
(A) Rates of coding sequence evolution. Distribution plots of *dN*/*dS* ratios of orthologous genes from bread wheat (W), *Ae. tauschii* (T), and *B. distachyon* (B). Data were fitted to a curve (red). Notes: *represents genes with at least one none-synonymous mutation and no synonymous mutations (*dN*/*dS* ratio = ≠0/0); **represents genes where the nucleotide and amino-acid sequences are identical (*dN*/*dS* ratio = 0/0). (For interpretation of the references to color in this figure legend, the reader is referred to the web version of this article.)

In order to identify genes which have undergone positive selection from *Ae. tauschii* to wheat, we assessed the ratio of non-synonymous-to-synonymous substitutions (*dN*/*dS)* for pairwise comparison of 136 orthologous genes considering wheat (W), *Ae. tauschii* (T) and *B. distachyon* (B) (Fig. 6). The distribution of *dN*/*dS* values in TW pairwise comparison was significantly different from those of BW and BT according to a Kolmogorov–Smirnov test (*p* < 0.001). In contrast to BW and BT, the pairwise comparison TW showed a *dN*/*dS* ratio distribution with a bias toward >0.5 values; additionally, 12.5% of the TW orthologs showed near identical nucleotide and amino-acid sequences (17/136 with *dN*/*dS* ratios ≠ 0), 14.7% showed some non-synonymous mutation and no synonymous mutation (20/136 with *dN*/*dS* ratio ≠ 0/0), and 20.6% showed identical nucleotide and amino-acid sequences (28/136 with *dN*/*dS* ratios = 0/0). We also examined the extremes of a total of 303 TW orthologous with gene ontology data and a first view suggested that there is no functional pattern associated with *dN*/*dS* ratios (see Table S4). The set of 14 genes with positive selection (*dN*/*dS* ratio > 1) included five with GO terms associated with biological processes, also five genes with cellular components GO terms, three with cellular components and one hypothetical protein without a GO term assigned. The opposite set of 28 orthologous genes with identical nucleotide and aminoacid sequences (*dN*/*dS* ratio = 0) included eight GO terms associated with biological processes, eight genes with molecular function, also eight genes with cellular components and four hypothetical proteins without GO terms assigned.

Supplementary Table S4 related to this article can be found, in the online version, at http://dx.doi.org/10.1016/j.plantsci.2014.12.004.

Supplementary Table S4 *dn*/*ds* ratios obtained from pairwise comparison of 136 orthologous genes considering wheat, *Ae. tauschii* and *B. distachyon*.

## 4 Discussion

Shotgun sequencing of flow-sorted chromosomes using NGS technologies provides a powerful approach to survey and assemble most of the gene space and low copy regions. Even though the technological robustness has been proved, the draft sequence of the large wheat genome might benefit from data provided by different platforms, such as Illumina and 454-pyrosequencing, since the genome sampling strategy holds differences between them. Interestingly, 4D chromosome GenomeZippers obtained with 454 data (our study) and Illumina [14] showed, in the case of 4DS, 639 shared syntenic loci, and remarkably, 252 (28%) 454-specific and 163 (20%) Illumina-specific loci. For 4DL, 849 shared syntenic loci were observed, combined with 232 (21%) 454-specific and 460 (35%) Illumina-specific loci. These data suggest a complementary coverage, at least in syntenic gene content, between 454 and Illumina data.

The GenomeZipper allows placement of the identified genes in the most probable order along the chromosomes, based on synteny [34,48,50–53]. The resulting gene order data may become a valuable marker source for mapping, positional gene cloning and physical map anchoring, especially in cases where traditional mapping suffers from a lack of resolution or polymorphism for marker development.

The 4D chromosome represents such a case. In several mapping studies this chromosome has been observed as carrying one of the lowest levels of polymorphism defined by linkage maps with few markers [17,18,35]. At least 20 genes/QTL associated with important agronomic traits have been identified on this chromosome, *e.g.* (i) fungal and viral resistance genes/QTL–*Qfhs.jic-4D*, *Lr22*, *Lr67*, *Yr28*, *Wsm1*, *Wss1* [54–59]; (ii) aluminum or salt tolerance genes/QTLs – *Alt2*, *Kna1* [60,61]; (iii) adaptation, flowering and yield-related genes/QTLs – *Vrn2*, *Rht1*, *QSpn.fcu-4D* [17,62,63,66,67], and (iv) quality-related genes – *Lpx1* [68,69].

Recent major advances in gene discovery and building of a highly saturated genetic map were achieved by *Ae. tauschii* genome survey sequencing [16]. In that study 30,697 protein coding genes were uniquely anchored to chromosomes with an integrated high density map including 2915 genes tagged at chromosome 4 obtained from 2568 scaffolds covering 180 Mb. Additionally, Luo et al. [19] reported an *Ae. tauschii* genome physical map spanning 520 Mb of chromosome 4 defined by 267 anchored contigs carrying 799 markers. The availability of comprehensive genomic datasets for *Ae. tauschii* chromosome 4 provides an excellent opportunity to thoroughly validate scaffolds/contigs belonging to *T. aestivum* chromosome 4D obtained by flow cytometry sorting.

In our study, the 4D assemblies created from low coverage of the flow-sorted chromosome arms provided sufficient quality and completeness to be the basis for the extensive analysis presented here. We have used content-based validation techniques to assess and quantify the biases introduced by the DNA preparation techniques, revealing good underlying sampling distributions based on known content.

Interestingly, from a total of 15,218 scaffolds, 7410 (49%) matched with *Ae. tauschii* scaffolds anchored to chromosome 4 and 5567 (37%) matched to unanchored *Ae. tauschii* scaffolds from Jia et al. [16]. As mentioned above, it is possible to speculate that some of these unanchored *Ae. tauschii* scaffolds belong to chromosome 4, but failed to be assigned in the mapping approach utilized by Jia et al. [16]. Remarkably, in that work ∼72% of the scaffolds larger than 1 kb could not be anchored to a particular *Ae. tauschii* chromosome. Accordingly, the 5567 4D scaffolds described in our work may serve as a complementary data resource to assign scaffolds and bridge gaps in the *Ae. tauschii* genome sequence.

Additionally, the identification of only 12% of scaffolds matching *Ae. tauschii* scaffolds anchored to chromosomes other than chromosome 4 corresponds well with the observed purity of the sorted chromosomal fractions we used in this study. This minimizes the limitations of sorted chromosomal fractions for survey sequencing using NGS approaches. Similar results have been reported for chromosomes 5A [44], 7DS and 7BS [51,52], 4A [48], 6B [64] and 5B [65].

Today, genomic approaches are probably one of the main drivers in biological science [71]. However, in addition to robust and relevant sequencing and assembly steps, achieving precise structural and functional genome sequence annotation is essential to provide the foundation for further relevant biological studies [72]. Therefore, whereas annotation is one of the most difficult tasks in genome projects, connecting genome sequence to biology is essential [73]. The modular architecture of the TriAnnot pipeline allows for the annotation and masking of transposable elements, the structural and functional annotation of protein-coding genes with evidence-based quality indexing, and the identification of conserved non-coding sequences and molecular markers [28]. In our study, modeling of protein-coding genes was performed using an improved version of the TriAnnot pipeline used for the annotation of wheat chromosome 3B [75] that enables (i) the automated validation of gene structure with the definition of high confidence (HC) and low confidence (LC) models, (ii) the identification of pseudogenes, and (iii) the exclusion of *ab initio* predictions without any similarity to plant proteins or with similarity only with TE-encoded proteins [75]. Using this approach 822 gene models were predicted (100 HC, 480 LC and 242 pseudogenes). The 100 HC gene models can be trusted and used for further analysis, while the 480 LC gene models may require additional manual curation, which is time consuming. The 242 pseudogenes may be revaluated since the short sequence length could in some cases explain the incomplete gene sequence in the scaffold, mainly at the 3′ end. It is believed that longer scaffold assemblies will greatly improve the quality of the wheat 4D chromosome annotation. Nevertheless, it is already promising that 381 TriAnnot-predicted genes were mapped on the GenomeZipper map. Moreover, 614 out of 822 gene models were functionally annotated by one or more GO terms.

A total of 1973 genes orthologous to the three reference genomes brachypodium, rice and sorghum, were predicted by the GenomeZipper approach. These genes were similarly distributed between both chromosome arms, with slightly more orthologous genes identified on 4DL. Noticeably, the proportion of orthologous genes that were supported by the three reference genomes was near 50% for 4DL and 25% for 4DS, suggesting that chromosome 4DS could have been more affected than 4DL by evolutionary changes. A linear gene order model for the entire wheat chromosome 4D was further constructed including wheat full length cDNA orthologous to Bd, Os and Sb, the public SNP map from *T. aestivum*, the wheat ESTs obtained from the HarvEST database (http://harvest.ucr.edu) and the gene models predicted by the TriAnnot pipeline resulting in 902 and 1092 loci for 4DS and 4DL, respectively, that could be virtually mapped. Thus, the gene maps of 4DS and 4DL were combined into one non-redundant map leading to a complete chromosome length of 98.81 cM, in which the centromere was located at 26.2 cM. On this map, even when the repetitive elements were distributed along both chromosome arms, the genes were mainly concentrated in the telomeric regions.

It is noticeable that there appeared to be very little synteny in the centromeric regions based on the *Ae. tauschii* genetic map and the 4D GenomeZipper data. However, bin-mapped ESTs indicated the presence of genes in this region, albeit at a lower level than in other regions of this chromosome. These findings agree with previous studies [70,76,77].

For both chromosome arms, a high level of collinearity was observed between the GenomeZipper and the *Ae. tauschii* chromosome 4 genetic map [16]. However, some inconsistencies were detected, mainly in the centromeric regions. These inconsistencies may reflect chromosomal rearrangements experienced by both species since the spontaneous hybridization event that occurred about 8000 years ago and gave rise to bread wheat. However, the possibility should be considered that they instead reflect misalignments in the wheat 4D GenomeZipper order and/or in the *Ae. tauschii* genetic map. The partial overlap of ESTs bin-mapped in deletion lines observed in some 4D regions support the first hypothesis.

As members of the same homoeologous group, chromosomes 4A, 4B and 4D share related genetic information with origin in the common ancestral species. Based on conserved syntenic gene loci, strong collinearity between 4D and 4B chromosomes was observed, in general terms. In agreement with previous reports, our results support that a structural change occurred in chromosome 4A involving a pericentric inversion [27,47,48]. It is difficult to make any conclusions from our data about minor rearrangements because low coverage, particularly for 4DL, may hinder the detection of syntenic conserved genes. Moreover, it is necessary to be cautious about the long-range structure of 4D–4B–4A Zippers herein described as it strongly depends on the backbone maps used. In the case of the D genome, the known low level of polymorphism (at least in comparison with A and B genomes) hinders to develop high density genetic maps and, in our study, a more detailed backbone of genetic markers anchored to the GenomeZipper map. Additional physical (bin-mapped markers) and genetic (*Ae. tauschii* map) data provided in our study (Figs. 4 and 5) should be valuable tools for choosing markers associated with a particular region of chromosome 4D. Moreover, the building of individual chromosome physical maps and gold standard sequences [72] and ultimately obtaining the reference sequence for the wheat genome will help overcome the described limitations.

### 4.1 Gene content

The syntenic genes identified by the GenomeZipper approach assessed in terms of sequence coverage, and the inclusion of non-syntenic genes (see Section 3) allowed the estimation of 5649 genes on chromosome 4D with an average density of 8.72 genes per Mb. This estimate is an intermediate value relative to results obtained for chromosomes 7BS (4.53/Mb) and 7DS (4.55/Mb) [51,52], 4A (5.02/Mb) [48], 6B (5.25/Mb) [64], 3AS (8.03/Mb) [78], 3B (9.62/Mb) [76] and 1BS (12.35/Mb) [70] (Table S5). According to the gene density values reported in these mentioned studies, it could be estimated that wheat genome comprise about 77,000–209,000 genes, if similar gene density values per chromosome can be assumed. However, in the case of the bread wheat genome, extrapolating results from NGS reads (or short contigs) may not reflect the overall genome structure and complexity, but this may be reduced by increasing the contig size [74] and/or through building and sequencing physical maps.

Supplementary Table S5 related to this article can be found, in the online version, at http://dx.doi.org/10.1016/j.plantsci.2014.12.004.

Supplementary Table S5 Gene density estimations per chromosome.

Further advances in massive sequencing and gene annotation of individual chromosome physical maps as well as improvements in LMP sequencing and forward analyses are required to solve these pending issues.

### 4.2 Rates of coding sequence evolution

We assessed the *dN*/*dS* rates for pairwise comparison of 136 orthologous genes (the set of 4D TriAnnot gene models), considering bread wheat (W) – *Brachypodium* (B) – *Ae. tauschii* (T) to identify genes with evidence of positive selection (*dN*/*dS* >v1) [41] and found 94–95% of BW and BT pairwise comparisons with less than 0.5 values (averages = 0.219 and 0.218, respectively). These data are in agreement with *dN*/*dS* values obtained from pairwise comparisons of 1022 orthologous sets of genes from chromosome 3A syntenic genes considering bread wheat, *Brachypodium* and rice (averages BW = 0.24, RW = 0.17, BR = 0.17) [79]. In contrast to previous data, WT gene pairs showed higher average (0.388) and a binomial distribution for the *dN*/*dS* ratios, with one peak at 0.2 and another peak at 0.6. About 13% of the genes showed a positive direction of selection (*dN*/*dS* > 0.5) (Fig. 6) suggesting that they might be under diversifying selection [80]. These data suggest a parallel (and opposite) evolution pattern of genes within chromosome 4D, defined by contrasting *dN*/*dS* ratios observed in WT orthologs, which unexpectedly show a different pattern defined by lower *dN*/*dS* ratios in the case of WB and BT orthologs, similar to that observed in previous data [16,79]. A similar behavior (parallel and contrasting evolution pattern) can be suggested for TW gene pairs carrying identical nucleotide and aminoacid sequences (*dN*/*dS* ratios = 0/0, 20.6%) and TW gene pairs with exclusively non synonymous mutations (*dN*/*dS* ratios = ≠0/0, 14.7%) (Fig. 6, see * and **).

**Supplementary Fig. S11:**
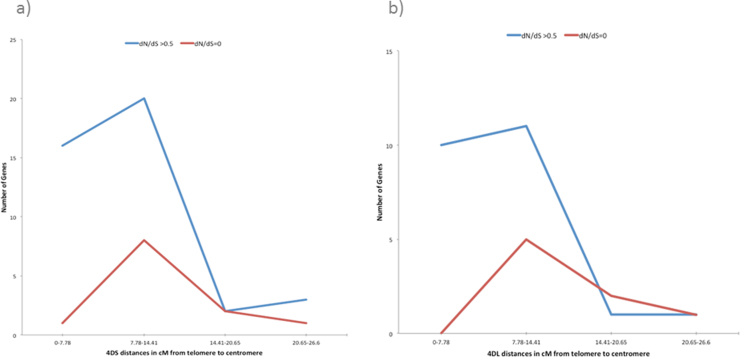
Distribution of the *dN*/*dS* ratios from orthologous genes along wheat chromosome arms 4DS (A) and 4DL (B) according to GenomeZipper virtual gene order. In blue, orthologous genes with *dN*/*dS* ratios >0.5, in red, orthologous genes with dN/dS ratios = 0.

We investigated the relationship between gene ontology terms and *dN*/*dS* ratios and did not detect specific association (Table S4). Alternatively, we analyzed the distribution of the *dN*/*dS* ratios along wheat chromosome 4D and our data showed a concentration of orthologous genes in the telomeres independent of *dN*/*dS* ratios (Fig. S11A and B), replicating the intrachromosomal density gene pattern described previously. In a third analysis, we calculated the average of *dN*/*dS* values obtained from BTW orthologs per chromosome arm, obtaining higher but non-significant values for 4DS (0.397) than for 4DL (0.356) (*P* = 0.6352).

Supplementary Fig. S11 related to this article can be found, in the online version, at http://dx.doi.org/10.1016/j.plantsci.2014.12.004.

## 5 Conclusions

Here we provide valuable information of wheat 4D chromosome obtained by pyrosequencing of both flow-sorted and multiple displacement amplified chromosome arms, available at NCBI (JROL00000000). A combination of different approaches using GenomeZipper, TriAnnot and EST bin maps allowed the prediction of 5649 genes on Chinese Spring 4D chromosome, comprising 2190 genes on 4DS and 3459 on 4DL. Comparison with other members of the wheat homeologous group 4 (4A and 4B) obtained from the IWGSC gave us an insight about the structure of this group of wheat chromosomes. The comparison showed higher level of collinearity between 4D and 4B compared to 4A, and confirmed that 4A underwent a pericentric inversion during bread wheat evolution. The resulting gene order data may become a valuable marker source for mapping, positional gene cloning and physical map anchoring, especially in cases where traditional mapping suffers from a lack of polymorphism for marker development, being an excellent tool for plant breeding programs.

## Conflict of interest

The authors declare that they have no competing interests.

## Authors’ contributions

CF, VE, MH, GT, NP conceived of the study. MH, NP, VE, GT, MC, JD, KM, PL participated in the study design and coordination. HS and MV prepared DNA. MR, BJC, MM, LV, SG performed bioinformatics analysis and contribute with scientific ideas. MH, GT, NP, VE and IG drafted the complete manuscript with contributions from the other authors. All authors read and approved the final manuscript.

## Figures and Tables

**Table 1:** Contig and scaffold assembly statistics from *Triticum aestivum* chromosome 4D.

Measures	Contigs				Scaffolds	
	Before repeat filtering		After repeat filtering			
	4DS	4DL	4DS	4DL	4DS	4DL
Total sequences	140,607	204,259	26,351	41,026	8141	7077
Total base pairs [Mbp]	103	120	34	36	38	27
Smallest length [bp]	100	100	100	100	1369	1530
Largest length [bp]	26,237	21,080	26,237	21,080	47,795	22,479
Average contig size [bp]	733.7	585.6	1287.8	868	4741	3817
L50 [bp]	1132	807	2314	1264	5517	3998

**Table 2:** Gene annotation of *Triticum aestivum* 4D scaffolds obtained using the TriAnnot pipeline. The number of genes in each category is given. HC = high confidence, LC = low confidence, CDS = coding sequence.

Chromosome Arm	HC full-length CDS	LC full-length CDS	Pseudogene	Total	Scaffold >6 kb
4DS	82	356	157	595	1771
4DL	18	124	85	227	840

**Table 3:** Genome zipper statistics. Number of markers, syntenic genes, contigs, wheat ESTs and full length cDNAs virtually mapped on *Triticum aestivum* chromosome 4D.

Data sets	4DS	4DL
Gene loci associated with genes from reference genomes	892	1081
*Brachypodium* genes	554	795
Rice genes	521	701
Sorghum genes	433	759
Wheat full length cDNAs	101	174
Wheat ESTs	289	409
Predicted genes	270	111
SNP markers	16	16
Total number of loci	902	1092
