# Supplementary material for: New insights into the wheat chromosome 4D structure and virtual gene order, revealed by survey pyrosequencing
Source: Plant Sci. 2015 Apr;233:200–12. doi: 10.1016/j.plantsci.2014.12.004 (PMC4352925; doi:10.1016/j.plantsci.2014.12.004)
Supplement: Supplementary Table S1 — Ae tauschii and wheat datasets used to assess 4D scaffolds. [file mmc5.docx]

**Supplementary Table 5. Gene density estimations per chromosome**

| **Chromosome/ chrom. arm** | **Size (Mb)** | **Gene number** | **Gene density (Mb)** | **Reference** |
| --- | --- | --- | --- | --- |
| 7BS | 360 | 1632 | 4.53 | Berkman et al. [27] |
| 7DS | 381 | 1732 | 4.55 | Berkman et al. [26] |
| 4A | 856 | 4300 | 5.02 | Hernandez et al. [28] |
| 6B | 914 | 4798 | 5.25 | Tanaka et al. [29] |
| 5A | 827 | 5088 | 6.15 | Vitulo et al. [25] |
| 3AS | 355 | 2850 | 8.03 | Sehgal et al. [84] |
| 4D | 648 | 5649 | 8.72 | this study |
| 3B | 18.2 | 175 | 9.62 | Choulet et al. [81] |
| 1BS | 314 | 3878 | 12.35 | Raats et al. [22] |
