# Supplementary material for: New insights into the wheat chromosome 4D structure and virtual gene order, revealed by survey pyrosequencing
Source: Plant Sci. 2015 Apr;233:200–12. doi: 10.1016/j.plantsci.2014.12.004 (PMC4352925; doi:10.1016/j.plantsci.2014.12.004)
Supplement: Supplementary Table S2 — Statistics of single end (SE) and long mate pair (LMP) reads used in this study. [file mmc1.docx]

| **Description** | **N seqs** | **Nucleotide total size** | **N50** | **Ref** |  |
| --- | --- | --- | --- | --- | --- |
|  |  |  |  |  |  |
| *Ae. tauschii* genome datasets | 429891 | 3020 Mb | 573 Kb | Jia et al. [16] |  |
| *Ae. tauschii* extended marker datasets | 6698 | 61 Mb | 10.8 Kb | Luo et al. [37] |  |
| 4D bin-mapped ESTs | 1057 | 0.5 Mb | 536 bp | Miftahudin et al. [43] | |

**Supplementary Table 1. Ae tauschii and wheat Datasets used to asses 4D scaffolds.**
