# Supplementary material for: New insights into the wheat chromosome 4D structure and virtual gene order, revealed by survey pyrosequencing
Source: Plant Sci. 2015 Apr;233:200–12. doi: 10.1016/j.plantsci.2014.12.004 (PMC4352925; doi:10.1016/j.plantsci.2014.12.004)
Supplement: Supplementary Table S3 — 4DS and 4DL Genome Zipper-based gene orders. [file mmc2.docx]

**Supplementary Table 2. Statistics of single end (SE) and long mate pair (LMP) reads used in this study**

|  | 4DL | 4DS | Total | # Runs |
| --- | --- | --- | --- | --- |
| **Reads** | 3174875 | 2981479 | 6156354 | **4 SE** |
| **Bases (bp)** | 1204121962 | 1132089628 | 2336211591 |  |
| **Length Average (bp)** | 379 | 380 | 379 |  |
| **Reads** | 1385664 | 1435833 | 2821497 | **2 LMP** |
| **Bases** | 508862344 | 534113457 | 1042975801 |  |
| **Length Average** | 367 | 372 | 369 |  |
| **Reads** | 4560539 | 4417312 | 8977851 | **TOTALS** |
| **Bases** | 1712984306 | 1666203085 | 3379187392 |  |
| **Length Average** | 373 | 376 | 374 |  |
